# Clinical Results of Carpal Tunnel Release Using Ultrasound Guidance in Over 100 Patients at Two to Six Years

**DOI:** 10.1016/j.jhsg.2024.02.004

**Published:** 2024-04-01

**Authors:** Logan C. Cano, Braeden M. Leiby, Laura C. Shum, Meliza G. Ward, Anthony E. Joseph

**Affiliations:** ∗ OrthoIdaho, LLC, Pocatello, ID; † Telos Partners, LLC, Warsaw, IN; ‡ Department of Family Medicine, Idaho State University, Pocatello, ID; § Department of Family Medicine, University of Washington, Seattle, WA

**Keywords:** Carpal tunnel release, Carpal tunnel syndrome, CTR-US, Minimally invasive surgery, Ultrasound

## Abstract

**Purpose:**

The purpose of this study was to determine the clinical results of carpal tunnel release using ultrasound guidance (CTR-US) at a minimum of 2 years postprocedure.

**Methods:**

The study consisted of 102 patients (162 hands) treated with CTR-US by the same physician between June 2017 and October 2020 for whom minimum 2-year follow-up data were available. Questionnaires were sent to gather long-term information, with additional phone calls for clarification if needed. Outcomes included Boston Carpal Tunnel Questionnaire symptom severity (BCTQ-SSS) and functional status (BCTQ-FSS) scores; Quick Disabilities of the Arm, Shoulder, and Hand (*Quick*DASH) scores; global satisfaction scores; and subsequent surgeries.

**Results:**

The 102 patients included 68 females and 34 males with a mean age of 56.9 years at the time of surgery. Fifty-five (53.9%) patients had simultaneous bilateral procedures, 42 (41.2%) had unilateral procedures, and 5 (4.9%) had staged bilateral procedures. Significant improvements in BCTQ-SSS, BCTQ-FSS, and *Quick*DASH scores persisted at a mean final follow-up of 46 months (range 2–6 years). At final follow-up, 91.2% of patients reported satisfaction with the procedure. No outcomes were significantly different between those treated with simultaneous bilateral versus unilateral procedures. No revision surgeries were reported.

**Conclusions:**

CTR-US is a safe and effective procedure that results in significant improvements that persist up to 6 years postprocedure. Long-term results of simultaneous bilateral and unilateral procedures are similar.

**Type of study/level of evidence:**

Therapeutic IV.

Carpal tunnel syndrome (CTS) is the most prevalent compression neuropathy in the United States, affecting 3% to 8% of the population.^1^, ^2^, ^3^, ^4^ In CTS, pressure on the median nerve may result in numbness, tingling, weakness, and pain in the hand and arm.^5^ Carpal tunnel release (CTR) is performed when nonsurgical interventions are ineffective, with over 600,000 procedures performed annually.^2^^,^^6^, ^7^, ^8^ Traditionally, CTR has been implemented through techniques such as open (OCTR), mini-open (mOCTR), and endoscopic (ECTR) procedures, all designed to transect the transverse carpal ligament (TCL) to relieve pressure on the median nerve and alleviate symptoms.^9^, ^10^, ^11^, ^12^, ^13^, ^14^ Recent advancements in surgical approaches have led to the development of CTR using ultrasound guidance (CTR-US), a minimally invasive technique that uses ultrasound to visualize critical structures while transecting the TCL through a small incision.^15^, ^16^, ^17^, ^18^, ^19^ Previously published studies, including randomized trials, have demonstrated the safety and efficacy of CTR-US as well as rapid clinical improvements attributed in part to the use of small, non-palmar incisions.^15^^,^^17^, ^18^, ^19^ Although multiple studies have demonstrated the durability of clinical improvements at 1 year post-CTR-US, only four publications by two author groups have reported longer-term outcomes for CTR-US at 2 years or more (Table 1).^15^, ^16^, ^17^^,^^20^, ^21^, ^22^, ^23^, ^24^, ^25^, ^26^, ^27^, ^28^, ^29^ Further research documenting the longer-term outcomes of CTR-US will help establish the relative durability of CTR-US compared with more commonly used techniques, such as OCTR, mOCTR, and ECTR (Table 1). Therefore, the primary purpose of the current study was to determine the clinical results of CTR-US at a minimum of 2 years postprocedure in a large group of over 100 patients. We hypothesized that at a minimum 2-year follow-up, patients treated with CTR-US would report significantly improved symptoms and function compared with baseline, high satisfaction with the procedure, and a rate of revision surgery that is similar to or less than that noted for more commonly used CTR techniques, such as OCTR, mOCTR, and ECTR.

**Table 1:** CTR Studies Reporting Clinical Results at Greater Than 2 Years of Follow-Up

Study	Study Design	Location	Follow-up (mean; range)	Technique	Patients	Hands
Atroshi et al^30^ (2009)∗†	RCT	Sweden	5 years	ECTR	63	NR
				OCTR	63	NR
Atroshi et al^31^ (2015)∗†	RCT	Sweden	12.8 years (SD: 1.2); range 11–16	ECTR	63	NR
				OCTR	61	NR
Fernández-de-las-Peñas et al^32^ (2020)‡	RCT	Spain	4 years	OCTR or ECTR§	48	NR
Keiner et al^33^ (2009)	Case series	Germany	8.2 years; range 5–12	ECTR	72	94
Louie et al^34^ (2013)	Retrospective case series	USA	13 years; 10–17	OCTR	113	113
Ly-Pen et al^35^ (2020)‡	RCT	Spain	6.3 years; range 6.1–9.8	mOCTR	NR	148
Malisorn^36^ (2023)	Retrospective cohort	Thailand	2 years	mOCTR	60	NR
				OCTR	60	NR
Nakamichi et al^37^ (2010)†	Prospective comparative	Japan	2 years	CTR-US	NR	63
Nakamichi and Tachibana^16^ (1997)	RCT	Japan	2 years	CTR-US	50	50
				OCTR	53	53
Schwarz et al^12^ (2022)	Retrospective comparative	Austria	60 months; range 36–108	mOCTR	50‖	50‖
			54 months; range 37–101	OCTR	50‖	50‖
Shah et al^38^ (2022)	Retrospective comparative	Pakistan	2 years	mOCTR	20	NR
				OCTR	20	NR
Wang et al^39^ (2019)	Prospective case series	Taiwan	2 years	CTR-US	84	113
Wang et al^40^ (2021)	Case series	Taiwan	2 years	CTR-US	376	628
Zhang et al^41^ (2016)	RCT	China	47 months (SD: 4.38)	CTR¶	73	73
			46 months (SD: 5.55)	OCTR	65	65
			46 months (SD: 6.27)	ECTR	69	69
Zhang et al^42^ (2019)	Retrospective cohort	USA	6.9 years; range 5.4–9.5	OCTR	12	NR
*Cano et al, 2024 (current study)*	*Retrospective cohort*	*USA*	*46 months; range 30–74*	*CTR-US*	*102*	*162*

NR, not reported. RCT, randomized controlled trial.

∗ Reports results from the same population.

† Includes bilateral surgeries.

‡ Study included other treatment arms in addition to CTR.

§ Based on physician and patient preference. Number of patients per treatment not reported.

‖ Number treated. Study did not report sample size at final follow-up.

¶ Double small incision CTR, introduced in this study.

## Methods

### Study participants

**Figure 1:**
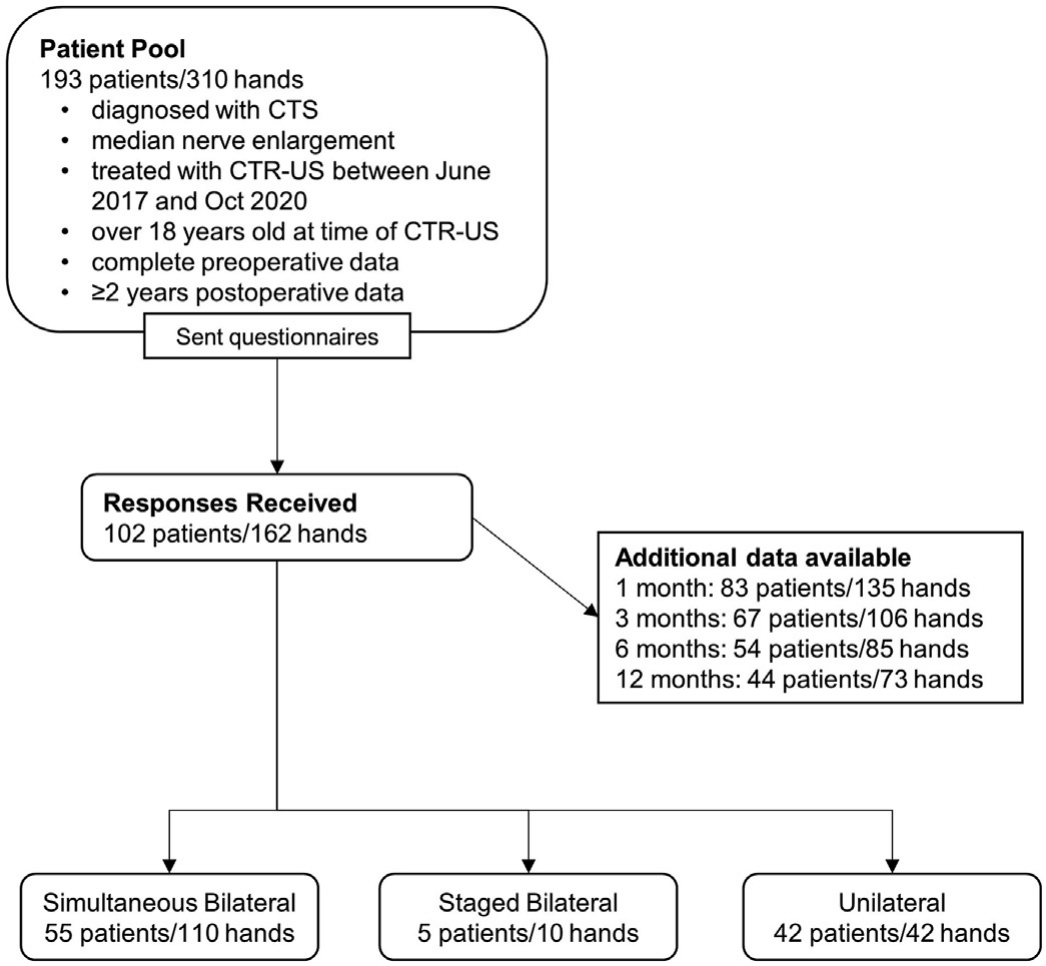
Flowchart showing patient selection and data availability. CTS, carpal tunnel syndrome; CSA, cross-sectional area; CTR-US, CTR using ultrasound guidance.

All patients who were (1) clinically diagnosed with CTS, (2) determined to have a median nerve enlargement on diagnostic ultrasound (cross-sectional area ≥10 mm^2^ or a wrist-pronator cross-sectional area of >2 mm^2^ for a non-bifid median nerve or >4 mm^2^ for a bifid median nerve), (3) treated with CTR-US by the senior author (A.E.J.) between June 2017 and October 2020 (ie, minimum 2 years postprocedure at the time of the study), and (4) over 18 years of age at the time of CTR-US were identified by chart review.^27^^,^^28^ Chart review found 193 patients (310 hands) who had complete preoperative data (see Patient-Reported Outcomes). These patients were contacted and asked to complete paper questionnaires administered by mail containing questions pertaining to their current CTS-related symptoms and function, satisfaction with the procedure, new conditions or diagnoses in the operated hand(s) (eg, nerve injury, recurrent CTS, etc.), and subsequent surgeries on the operated hand(s). Responses were received from 102 patients (162 hands, 52.8%, Fig. 1). Patients responding affirmatively to the questions pertaining to new conditions, diagnoses, or subsequent surgeries on the operated hand(s) were contacted by phone to obtain additional information, specifically to determine whether there was evidence of a complication potentially related to the CTR-US procedure, including revision CTR. Boston Carpal Tunnel Questionnaire (BCTQ) and Quick Disabilities of the Arm, Shoulder, and Hand (*Quick*DASH) scores prospectively collected from the same patients preoperatively and at routine follow-up appointments were compared with the BCTQ and *Quick*DASH results obtained from the long-term patient questionnaires. For the 102 patients (162 hands), BCTQ and *Quick*DASH results collected as part of routine postoperative care were available for 83 patients (81.4%) at 1 month, 67 patients (65.7%) at 3 months, 54 patients (52.9%) at 6 months, and 44 patients (43.1%) at 12 months. Clinical results from a subset of these patients at 3 months and 1 year post-CTR-US have been previously reported.^15^^,^^29^ All participants included in the study provided written informed consent. The study was conducted per the Declaration of Helsinki and Strengthening the Reporting of Observational Studies in Epidemiology (STROBE) guidelines.^43^ This study was approved by the Idaho State University Human Subjects Committee (FWA 00014037).

### Procedural description

**Figure 2:**
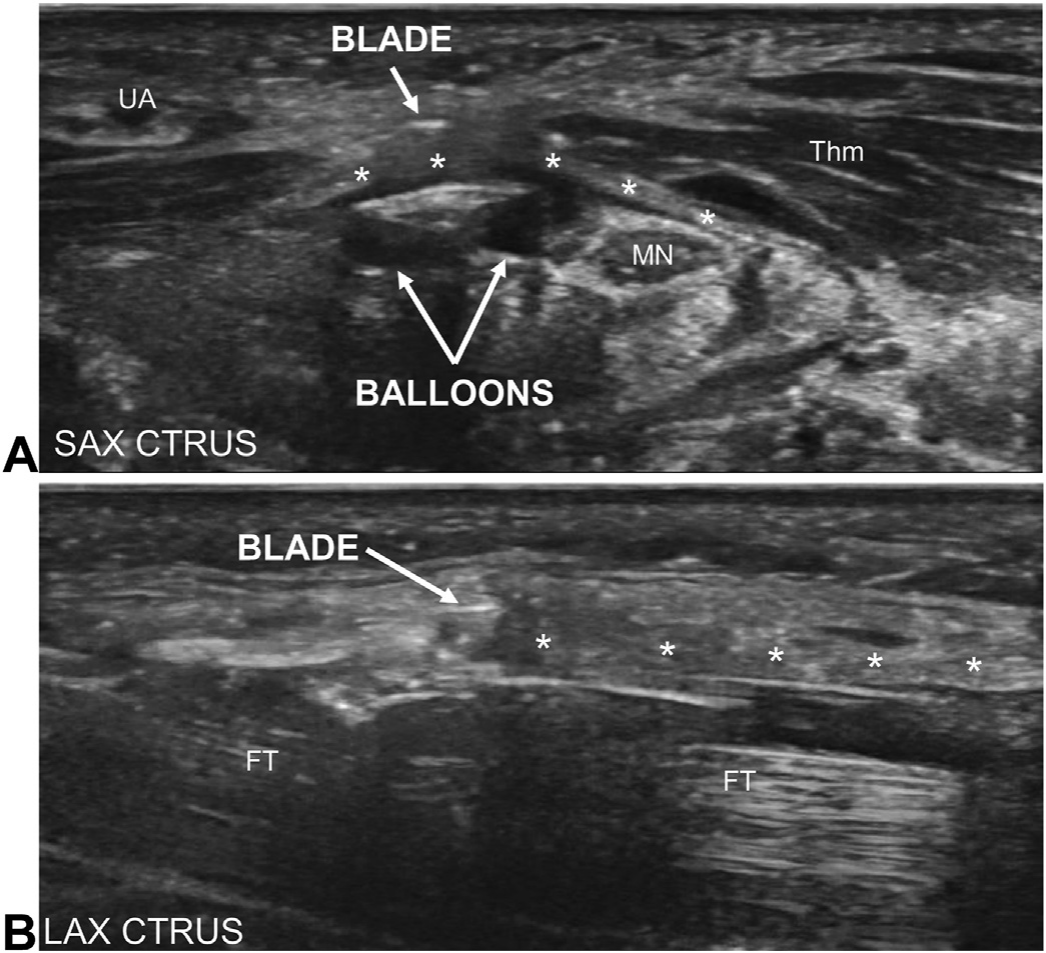
Carpal tunnel release with ultrasound guidance. **A** Transverse view of the distal carpal tunnel (left = ulnar) showing the blade tip of the device superior to the transverse carpal ligament (TCL, asterisks) and between the ulnar artery (UA) and the median nerve (MN). The balloons were deployed to create space and separate the MN from the centrally located blade. **B** Longitudinal view (left = distal) showing the device directly under the TCL with the cutting blade visible and engaged to transect the TCL. Thm, thenar muscles.

All CTR-US procedures were performed by the senior author in a hospital operating room or ambulatory surgery center between June 2017 and October 2020. All procedures were performed under ultrasound guidance and local anesthesia, with anxiolytic medication (midazolam 2-4 milligrams intravenously) per patient preference. Details of the procedure have been previously described.^15^^,^^29^ In brief, the carpal tunnel was scanned with ultrasound to identify key anatomical landmarks (eg, median nerve, ulnar artery and superficial arch, digital nerves, thenar motor branch, etc.), and then a small longitudinal incision, typically less than 5 mm, was made in the region of the proximal wrist crease. CTR-US was performed using a commercially available device (UltraGuideCTR, Sonex Health, Inc.) that creates space in the carpal tunnel while protecting neurovascular structures using two inflatable balloons. The device was positioned in the carpal tunnel using ultrasound, and the balloons were inflated. Then, the retrograde cutting blade was deployed to transect the TCL under continuous ultrasound guidance (Fig. 2). After transection, the blade was recessed, balloons were deflated, and the ligament was probed using ultrasound visualization to ensure a complete release. Wounds were typically closed with sterile adhesive wound closure strips (ie, no sutures) and dressed with sterile gauze, sterile transparent film dressing, and a compression wrap. Patients received instruction on edema control and use of nonsteroidal anti-inflammatories or acetaminophen for postoperative pain management.

### Patient-Reported Outcomes

Clinical outcomes assessed by questionnaire included the Boston Carpal Tunnel Questionnaire symptom severity and functional status scores (BCTQ-SSS and BCTQ-FSS, respectively), the *Quick*DASH score, a Global Satisfaction Score (five-point scale from 1 = very dissatisfied to 5 = very satisfied), and subsequent conditions, diagnoses, or surgeries on the operated hand(s). The BCTQ is a CTS-specific questionnaire with questions on symptom severity and functional status.^44^ Scores for BCTQ-SSS and BCTQ-FSS range from 1 to 5, with higher scores indicating more severe symptoms or functional limitations, respectively. *Quick*DASH is a patient-reported questionnaire with a total score ranging from 0 (no disability) to 100 (severe disability).^45^ BCTQ-SSS score was obtained independently for each hand for patients treated with simultaneous bilateral releases. *Quick*DASH, BCTQ-FSS, and satisfaction was assessed separately for each hand. Minimal clinically important differences (MCIDs) for postoperative change in patient-reported outcomes were considered to be 1.14 points for BCTQ-SSS, 0.74 points for BCTQ-FSS, and 15 points for *Quick*DASH.^46^^,^^47^ Patients were asked about additional diagnoses on their operated hand(s) based on the question “Has any health care provider diagnosed you with the following in your CTR hand?”. Possible answers were (a) nerve injury, (b) vessel injury, (c) infection, (d) recurrent CTS, and (e) none. Patients were also asked if they had any subsequent injuries or surgeries on the operated hand(s). We attempted to contact all patients with an affirmative response to obtain additional information, specifically to determine whether there was evidence of a complication potentially related to the CTR-US procedure, including revision CTR. Responses that were determined to be in error were removed from analysis.

### Statistical analysis

At each reported time point, BCTQ-SSS, BCTQ-FSS, *Quick*DASH, and satisfaction data were summarized using the mean and standard deviation of the mean (SD) unless otherwise specified. Final follow-up was calculated as the mean amount of time between CTR-US procedure and the date the questionnaire was completed. For BCTQ and *Quick*DASH, pre- and postoperative scores were calculated and summarized using means and standard error of the mean (SEMs). Differences were assessed using a two-tailed *t*-test unless otherwise specified. For the simultaneous bilateral versus unilateral procedure comparison, differences were assessed using the Mann–Whitney test. *P* < .05 was considered statistically significant.

## Results

**Figure 3:**
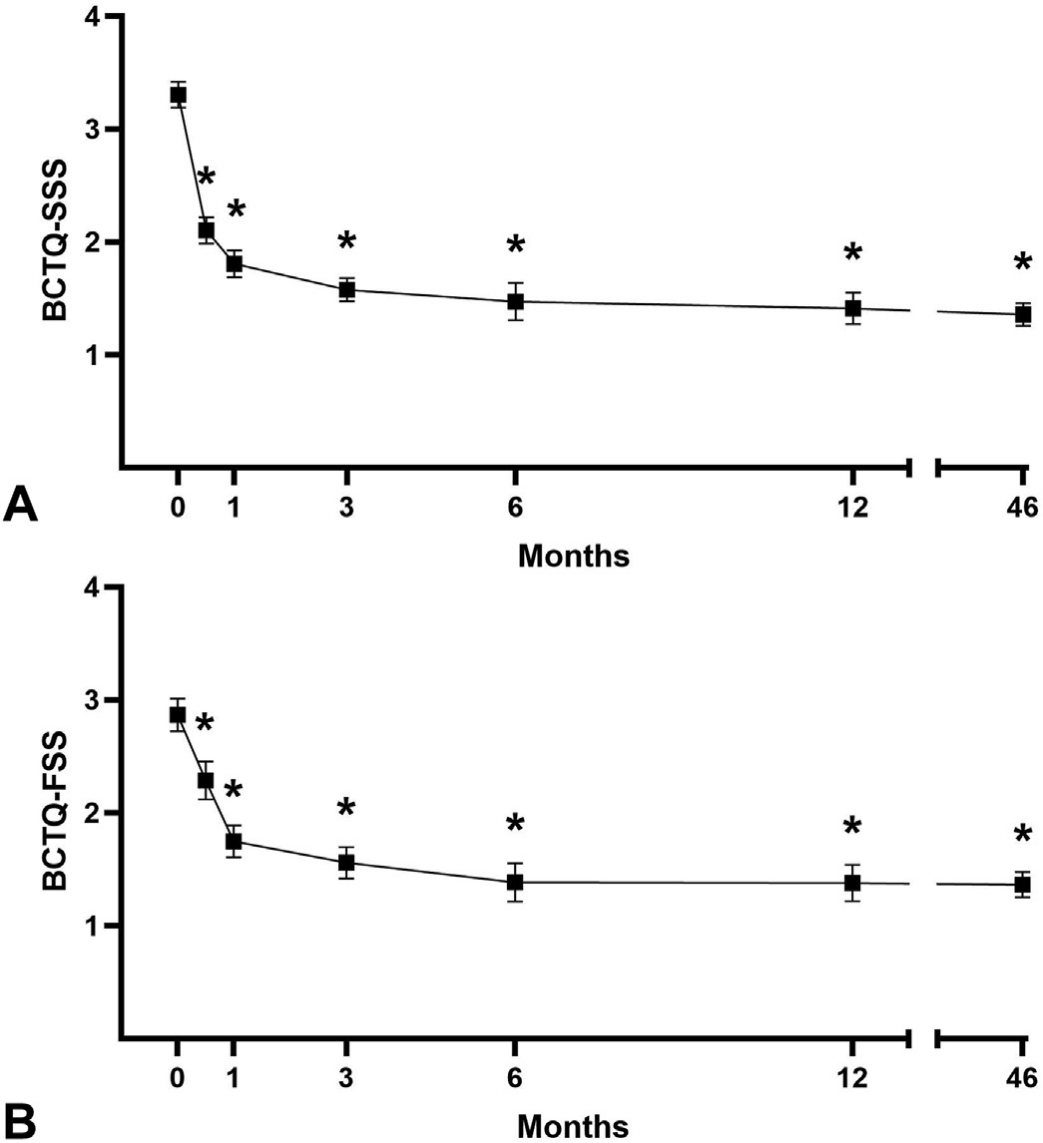
Mean BCTQ scores ± SEM over time post-CTR-US. Improvements in BCTQ-SSS and BCTQ-FSS scores are maintained through a mean follow-up of 46 months (2–6 years). ∗*P* < .0001 compared with preoperative baseline score. See Methods for sample size at each timepoint.

**Figure 4:**
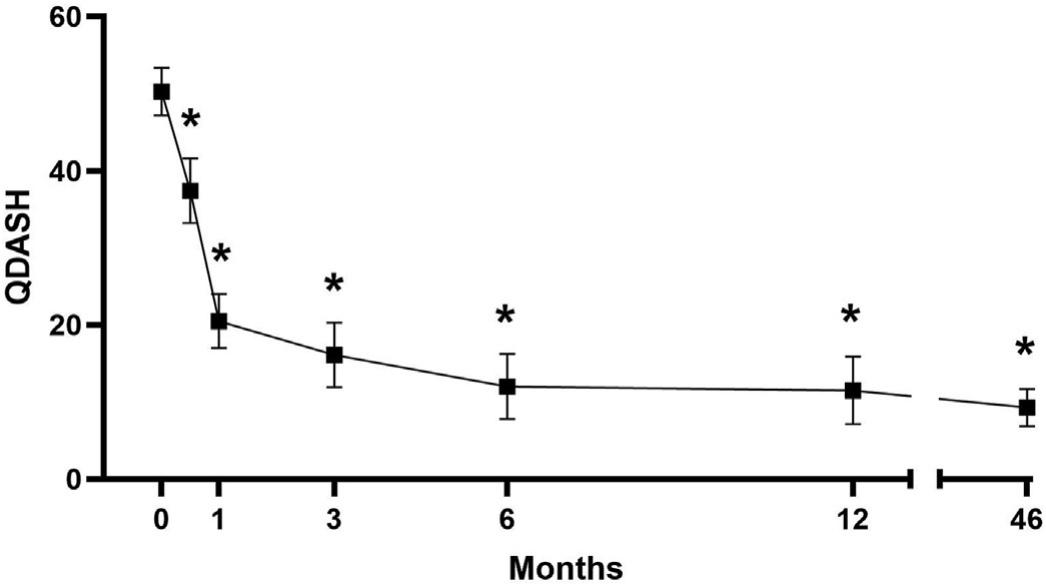
Mean *Quick*DASH ± SEM over time post-CTR-US. Improvements in *Quick*DASH scores are maintained through a mean follow-up of 46 months (2–6 years). ∗*P* < .0001 compared with preoperative baseline score. See Methods for sample size at each timepoint.

Patient demographics were typical of a CTS population (Table 2), and the mean follow-up was 46 months (range 30–74, Table 3). Mean BCTQ-SSS scores were 3.3 ± 0.7 at baseline, 2.1 ± 0.6 at 0.5 months, 1.4 ± 0.6 at 12 months, and 1.4 ± 0.6 at final follow-up (*P* < .001, all timepoints vs baseline, Fig. 3). Mean BCTQ-FSS scores were 2.9 ± 0.9 at baseline, 2.3 ± 0.9 at 0.5 months, 1.4 ± 0.7 at 12 months, and 1.4 ± 0.7 at final follow-up (*P* < .001, all timepoints vs baseline, Fig. 3). Mean *Quick*DASH scores were 50.9 ± 20.1 at baseline, 38.5 ± 24.1 at 0.5 months, 11.7 ± 18.2 at 12 months, and 9.5 ± 15.7 at final follow-up (*P* < .001, all timepoints vs baseline, Fig. 4). Improvements in patient-reported outcomes surpassed reported MCIDs.^46^^,^^47^ Patient satisfaction was consistently high at 12 months and final follow-up, with 91.2% of patients reporting satisfaction (Table 4).

**Table 2:** Patient Characteristics

Characteristic	Patients (n = 102)	
	n	(%)
Age (y)		
Mean (SD)	56.9	(14.4)
18–34	8	7.8%
35–64	63	61.8%
≥65	31	30.4%
Sex		
Women	68	66.7%
Men	34	33.3%
Operative Hand		
Dominant only	20	19.6%
Nondominant only	6	5.9%
Not reported	16	15.7%
Procedure		
Simultaneous bilateral	55	53.9%
Staged bilateral	5	4.9%
Unilateral	42	41.2%
Baseline Symptoms and Function	Mean (SD)	
BCTQ-SSS (1–5 scale)	3.3	(0.7)
BCTQ-FSS (1–5 scale)	2.9	(0.9)
*Quick*DASH (0–100 scale)	50.9	(20.1)

**Table 3:** Duration of Final Follow-Up

Follow-up (months)		
Mean ± SD	46.0 ± 10.9	
Median	44.9	
Range	30.1–74.0	
Distribution	Patients	Hands
≥2 years	102	162
≥3 years	78	127
≥4 years	41	69
≥5 years	13	21

**Table 4:** Patient Satisfaction

Satisfaction	12 months n = 73 hands	46 months n = 162 hands
Score (mean ± SD)∗	4.6 ± 0.9	4.6 ± 0.9
% Satisfied†	87.0%	91.2%

∗ Scale from 1 (very dissatisfied) to 5 (very satisfied).

† Percent satisfied calculated as number of hands with a Satisfaction Score ≥ 4 divided by total number of procedures per time period.

On the long-term questionnaire, patients were asked if they had any new diagnoses or conditions on their operated hand(s) subsequent to their CTR-US procedure (see Methods). Three patients (five hands) reported “nerve injury,” and two patients (four hands) reported “recurrent CTS.” When contacted for additional information, one patient (one hand) who reported “nerve injury” had not been diagnosed with a nerve injury but had temporary neuritis symptoms that subsided without treatment. Another patient (two hands) reporting “nerve injury” had not been diagnosed with a nerve injury; this patient had a pre-existing sensorimotor neuropathy that did not improve following CTR-US and reported no subsequent surgeries at 3.7 years follow-up. The third patient (two hands) reporting “nerve injury” could not be reached for additional information, but this patient’s questionnaire at 3.2 years post-CTR-US indicated improvement in symptoms, no subsequent surgeries, and a satisfaction score of five out of five for both hands. The two patients who reported “recurrent CTS” were also contacted. One patient clarified that they had received subsequent bilateral surgery for de Quervain’s tenosynovitis and did not believe their symptoms were attributed to recurrent CTS. The second patient was diagnosed with cervical spondylosis, and symptoms subsided after cervical disc replacement. Twelve patients reported subsequent surgeries or major injuries on the operated hand(s). Five had trigger finger release, and the remaining surgeries or injuries included anterior cervical discectomy fusion (a different patient than above), arthritis, broken finger, joint replacement, motor vehicle accident, severed tendon attributed to an animal-related incident, and stent placement with a right wrist approach. No revision carpal tunnel surgeries were reported.

We found no difference in the long-term clinical outcomes between patients treated with simultaneous bilateral versus unilateral CTR-US procedures. Staged bilateral procedures were excluded from this comparison given the low number of procedures (n = 5, Table 2). Mean change from baseline to final follow-up for BCTQ-SSS, BCTQ-FSS, and *Quick*DASH scores were not significantly different between unilateral and simultaneous bilateral procedures (Table 5), and no difference was found between groups with respect to satisfaction at final follow-up (90% simultaneous bilateral; 93% unilateral). Differences did not surpass MCID for any outcomes.

**Table 5:** Improvements at Final Follow-up: Simultaneous Bilateral Versus Unilateral Procedures

Procedure	BCTQ-SSS	BCTQ-FSS	*Quick*DASH
Simultaneous bilateral	−1.9 ± 1.0	−1.5 ± 1.0	−42.0 ± 23.4
Unilateral	−1.9 ± 0.8	−1.3 ± 1.1	−37.5 ± 17.6
*P* value∗	0.9780	0.3939	0.2753

*P* value calculated using the Mann–Whitney test.

## Discussion

This study demonstrates the long-term durability of positive outcomes following CTR-US, extending our previous findings.^15^^,^^29^ At a mean of almost 4 years post-CTR-US, patients continued to report significant and clinically meaningful improvements in symptoms and function with high satisfaction. Importantly, clinical results were essentially equivalent between 1 year post-CTR-US and the final follow-up at 2–6 years, a finding consistent with long-term studies of other commonly used CTR techniques, such as OCTR and ECTR (Table 1). In summary, the current investigation supports and expands upon the published literature reporting the long-term safety and effectiveness of CTR-US, with no differences between simultaneous bilateral and unilateral procedures.

Few have reported results beyond 1 year post-CTR-US. Here, we report on 102 patients (162 hands) at a mean follow-up of 46 months, with a range of 30–74 months, including 13 patients (21 hands) with greater than 5 years of follow-up (Table 3). Only four previous studies (2 groups) have reported 2-year results of CTR-US, only one included more than 100 patients, and none had follow-up beyond 2 years.^16^^,^^37^^,^^39^^,^^40^ Consequently, the current results address an important gap in the literature with respect to the durability of CTR-US results at 2 years and beyond. Our results are consistent with CTR-US studies from Nakamichi et al^16^^,^^37^ and Wang et al^39^^,^^40^ as well as longer-term results from other CTR techniques, such as OCTR and ECTR.^12^^,^^30^, ^31^, ^32^, ^33^, ^34^, ^35^, ^36^^,^^38^^,^^41^^,^^42^ Of note, we had no reports of revision CTR for recurrent symptoms, similar to the other long-term CTR-US studies.^16^^,^^37^^,^^39^^,^^40^ Given that the historical rate of revision for recurrence is approximately 0.5% to 1% for OCTR and ECTR, the lack of revision is noteworthy.^9^^,^^48^ Additionally, over 90% of patients in the current investigation reported satisfaction with the procedure, and patient satisfaction was stable between 1 year and the final follow-up (87.0% at 1 year vs 91.2% at final, Table 4).

Strengths of this study include the large number of patients (Table 1) and the extended follow-up duration (Table 3). Additionally, more than half (54%) of the surgeries were simultaneous bilateral procedures. However, this study also has potential limitations to consider. First, all procedures were performed by a single, experienced physician (>6 years of experience with CTR-US and >250 procedures), which may limit generalizability. However, our results are consistent with previous studies reporting 1-year results by a diverse group of physicians using the same technique in a typical CTS patient population.^20^^,^^25^ Second, not all eligible patients (ie, those who were minimum 2 years post-CTR-US), participated in the study, introducing the potential for selection bias. Nonetheless, the response rate of over 50% is noteworthy for a study of this size with a minimum 2-year follow-up. Third, the long-term data for this study were collected using paper questionnaires, with follow-up phone calls as needed. This methodology could have precluded information that may have been acquired in-person.

In conclusion, the current study demonstrates the long-term durability of positive outcomes following CTR-US at a mean of 46 months accompanied by a high level of patient satisfaction. These results are comparable to long-term data reported for open and endoscopic CTR.

## Conflicts of Interest

No benefits in any form have been received or will be received related directly to this article.
